# More Than Meets the Eye: Infective Endocarditis Presenting as Endogenous Endophthalmitis

**DOI:** 10.7759/cureus.14745

**Published:** 2021-04-28

**Authors:** Ricardo J Villasmil, Natalia Lattanzio, Katherine Burns, Talal Alkayali

**Affiliations:** 1 Internal Medicine, Florida State University College of Medicine Internal Medicine Residency Program, Sarasota, USA

**Keywords:** viridian's group endocarditis, aortic valve abscess, acute endophthalmitis, bacteremia, streptococcal bacteremia, aortic valve

## Abstract

Endogenous endophthalmitis (EE) is a potentially blinding ophthalmological emergency with poor visual prognosis requiring a high index of clinical suspicion to obtain a prompt diagnosis. The main factors associated with poor visual outcomes include the virulence of the causative organism and timing of intervention. Infective endocarditis can present with nonspecific ocular complaints and remains an important risk factor for developing EE. We report an unusual case of EE in a 78-year-old male caused by infective endocarditis secondary to *Streptococcus viridans* resulting in bioprosthetic aortic valve abscess with dehiscence.

## Introduction

Endophthalmitis is defined as an intraocular infection of aqueous or vitreous humor caused by bacteria or fungi. The infection can be further categorized into exogenous and endogenous. Most reported cases involve exogenous endophthalmitis resulting from the introduction of the causative organism during ocular surgery or via penetrating trauma [1]. Endogenous endophthalmitis (EE) is a rare but potentially sight threatening condition caused by hematogenous spread of bacteria from a remote primary source and accounts for 2% to 8% of all cases of endophthalmitis [2].

Aetiologic organisms differ by geographical region. In the United States, gram-positive organisms such as *Staphylococcus aureus* and *Streptococcal *species are the most common [3]. Common predisposing risk factors include diabetes mellitus, chronic immunosuppression and endocarditis. Although cardiac complications of endocarditis are more prevalent, ocular manifestations are nonspecific and could be the first manifestation of disease [4]. We report a case of a 78-year-old male presenting with EE caused by infective endocarditis secondary to *Streptococcus viridans *resulting in bioprosthetic aortic valve abscess with dehiscence.

## Case presentation

A 78-year-old male with history of myelodysplastic syndrome (MDS), dementia and a previous episode of endocarditis 10 years prior which required bioprosthetic aortic valve replacement, presented with a three-day history of progressive left eye vision loss accompanied with floaters. He denied chest pain, shortness of breath or fever. At presentation, vital signs were a heart rate of 90 bpm, blood pressure 129/56 mmHg, temperature of 98.2°F and respiratory rate of 17 rpm. Pertinent physical exam findings consisted of a grade II/VI systolic ejection murmur at the left sternal border. There were no peripheral signs suggestive of endocarditis such as Osler’s nodes, Janeway lesions, or splinter hemorrhages. Ophthalmic examination of the left eye revealed a visual acuity of 20/30 and intraocular pressure by tonopen of 8 mmHg with pupils equal and reactive to light. There was evidence of conjunctival injection with dilated episcleral vessels and scattered retinal hemorrhages with cells obscuring the posterior chamber. The results of external, slit-lamp, and fundus examinations of the right eye were normal. Laboratory data was significant for a leukocytosis of 20,300 with left shift and a thrombocytopenia of 27,000.

Following ophthalmologic evaluation, given the high concern for EE, he underwent emergent left eye vitrectomy with vitreous sample and culture. He received prophylactic intravitreal antibiotics with vancomycin, ceftazidime and amphotericin B as well as systemic intravenous vancomycin and voriconazole. Both blood and vitreous cultures grew *Streptococcus viridans.* He was diagnosed with left eye endogenous endophthalmitis and started on ceftriaxone according to bacterial sensitivities for a planned 42-day intravenous antibiotic course. He underwent further evaluation with transthoracic echocardiogram to assess for the primary source of infection which demonstrated an ejection fraction of 60%, grade 1 diastolic dysfunction and findings suggestive of aortic valve regurgitation without evidence of vegetation. Five days after initiation of antibiotics, a transesophageal echocardiogram revealed severe aortic regurgitation with severe bioprosthetic aortic valve abscess with dehiscence along the aorto-mitral curtain (Videos 1-2). The patient’s hospital stay was complicated with worsening thrombocytopenia from 27,000 to 12,000 not responsive to multiple platelet transfusions raising concern for superimposed drug-induced thrombocytopenia. The patient’s antibiotic regimen was changed from ceftriaxone to vancomycin with no improvement in platelet count. A bone marrow biopsy was performed which revealed worsening MDS. On his fourth day of hospitalization, he developed acute hypoxic respiratory failure secondary to new onset acute diastolic congestive heart failure requiring transfer to the intensive care unit. A pro-BNP was obtained and elevated at 30,000 pg/dL. He was evaluated by cardiothoracic surgery for aortic valve replacement and deemed a poor surgical candidate secondary to critically ill condition, thrombocytopenia and advanced dementia. The patient and his wife ultimately elected to pursue hospice care.

**Video 1:** Transesophageal echocardiogram (TEE) mid-esophageal aortic valve short axis view of bioprosthetic aortic valve abscess.

**Video 2:** Transesophageal echocardiogram (TEE) mid-esophageal aortic valve long axis view showing severe aortic valve regurgitation and vegetation.

## Discussion

Infective endocarditis (IE) is an infection of the endocardium of the heart which may include one or more heart valves. Intravenous drug use and prosthetic heart valves remain important risk factors as prosthetic valve endocarditis accounts for 20% of all cases of endocarditis [5]. IE can result in valvular destruction and perivalvular abscess formation which often can result in valvular dehiscence and acute heart failure. However, friable infected tissue can spread hematogenously to other areas resulting in bacterial seeding. A review of 72 cases of metastatic endophthalmitis revealed 10 cases to be secondary to endocarditis with the majority of the cases involving both eyes [6]. Ocular manifestations of endocarditis are nonspecific with the majority of manifestations being a result of microembolization. Roth spots have been classically associated with endocarditis but are nonspecific and can be seen with various medical conditions including blood dyscrasias and vasculitides [7]. Although originally claimed to be present in 80% of IE cases, recent research has shown only 2% of patients actually have Roth spots present and thus clinicians must carefully examine for other endocarditis stigmata [8]. While cardiovascular complications are the most common in IE, clinicians should be aware of extracardiac manifestations which may result from septic embolism including acute endophthalmitis.

EE is a rare ocular manifestation of IE with high mortality rates and poor visual prognosis that poses a difficult diagnostic challenge. Identifying the primary source of infection should be promptly sought. As IE is the second most common cause of EE after meningitis, a transesophageal echocardiogram should be obtained if suspicion for endocarditis is high as a transthoracic echocardiogram is not recommended to rule out endocarditis. Vitreous fluid culture has the highest yield in identifying the causative organisms compared to blood cultures, 87% to 33% respectively [9]. In the United States and Europe, IE is most common due to gram positive organisms [10]. *Streptococcus viridans* is an avirulent organism which has been implicated in causing endophthalmitis and subacute endocarditis but unlike the case presented, it has rarely been associated to cause valvular destruction or perivalvular abscess [11]. The subacute endocarditis caused by viridans streptococci is characterized by an indolent disease course. However, the presence of *Streptococcus viridans* bacteremia should raise concern for IE particularly in individuals with risk factors such as previous history of endocarditis and IV drug use.

Treatment should be initiated as soon as EE is suspected before obtaining confirmatory cultures. The mainstay of treatment involves intravitreal therapy, systemic antibiotics and in some circumstances vitrectomy. Ceftazidime and vancomycin are the preferred intravitreal antibiotics for bacterial EE. Amphotericin B is added if fungal infection is suspected [11]. In choosing systemic antibiotics, clinicians should keep two important factors in mind, ocular penetration and breadth of coverage as determined by the suspected source of infection [12].

## Conclusions

Endogenous endophthalmitis is an ophthalmological emergency that could be the first sign of infective endocarditis. This case highlights the importance of maintaining a high index of suspicion for infective endocarditis in patients presenting with endophthalmitis despite normal transthoracic echocardiography. Early diagnosis and administration of intravitreal antibiotics and surgical evaluation remains the cornerstone of therapy for endogenous endophthalmitis.
